# Isostreptazolin and Sannaphenol, Two New Metabolites from *Streptomyces sannanensis*

**DOI:** 10.3390/molecules17010836

**Published:** 2012-01-16

**Authors:** Dan Zheng, Li Han, Yiqing Li, Jun Li, He Rong, Qiao Leng, Yi Jiang, Lixing Zhao, Xueshi Huang

**Affiliations:** 1 Ministry of Health Key Laboratory of Congenital Malformation, Laboratory of Metabolic Disease Research and Drug Development, China Medical University, Shenyang 110001, China; 2 Key Laboratory of Microbial Diversity in Southwest China, Ministry of Education, Yunnan University, Kunming 650091, China

**Keywords:** isostreptazolin, sannaphenol, *Streptomycessannanensis*

## Abstract

Two new compounds, isostreptazolin (**1**) and sannaphenol (**2**), were isolated from the culture broth of *Streptomyces sannanensis* and their structures elucidated on the basis of 1D and 2D NMR as well as MS, IR and UV spectroscopic data analysis. The cytotoxic activity of **1** and **2** were evaluated. Both compounds were inactive against H460 and HeLa cell lines at 100 μM.

## 1. Introduction

In the course of screening for new bioactive compounds from microbial sources, two new constituents, isostreptazolin (**1**) and sannaphenol (**2**), were isolated from the fermentation broth of *Streptomyces sannanensis*, from which a new cytotoxic alkaloid, sannanine, had been isolated by the authors in earlier work [1]. The structures of **1** and **2** were determined by detailed spectroscopic investigation and the results of biological testing for the cytotoxic activity of **1** and **2** are presented. The remarkable structural feature of **1** is a unique tricyclic skeleton with a urethane moiety of natural origin. The unusual ring system is rarely seen in Nature and only found in streptazolin [2,3] and its analogues [4,5]. The biosynthesis of streptazolin had been discussed by M. Mayer in 1993 [6]. As streptazolin and **1** bear analogous structural elements, both of compounds possibl originate from a similar biosynthetic pathway or derive from the same biosynthetic polyketide precursor.

**Figure 1:**
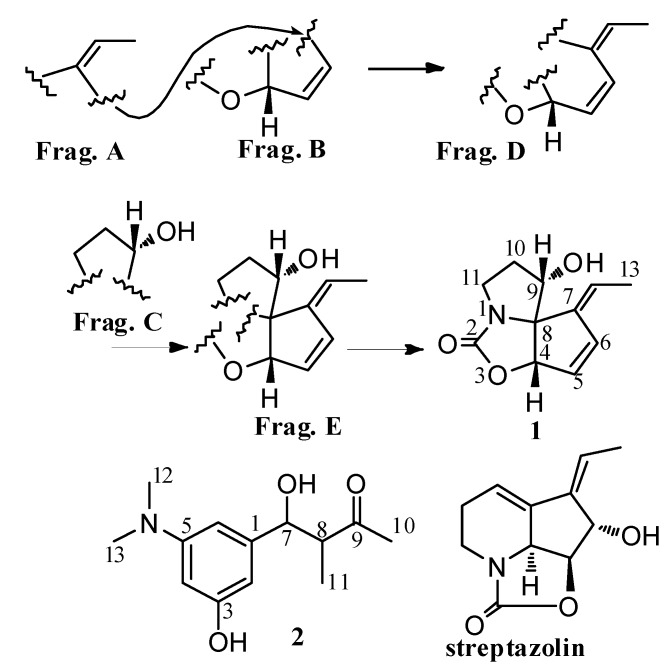
Structures of compound **1**, **2**, and streptazolin.

## 2. Results and Discussion

### 2.1. Chemistry

The molecular formula of **1** was established as C_11_H_13_NO_3_ by HRESI-MS. The IR spectrum of **1** indicated the presence of a hydroxy group (3366 cm^−1^) and a carbonyl group (1718 cm^−1^). The ^1^H- NMR spectrum of **1** (Table 1) showed three olefinic proton signals at *δ* = 6.80 (1H, d, *J* = 5.8 Hz), 6.21 (1H, brd, *J* = 5.8 Hz), and 5.44 (1H, q, *J* = 7.2 Hz), two oxygenated methine signals at *δ* = 5.50 (1H, d, *J* = 1.8 Hz), 4.11 (1H, brt, *J* = 4.0 Hz), two methylene signals at *δ* = 3.58 (1H, ddd, *J* = 11.0, 9.2, 7.3 Hz), 3.11(1H, ddd, *J* = 11.0, 9.9, 4.0 Hz) and *δ* = 2.48 (1H, m), 2.05 (1H, m). Moreover, a methyl signal at *δ* = 1.75 (3H, d, *J* = 7.2 Hz) and an active proton signal at *δ* = 5.47 (1H, d, *J* = 4.0 Hz) were observed. The ^13^C-NMR spectrum of **1** (Table 1) indicated the carbon atoms corresponding with the ^1^H NMR data. In addition, three quaternary carbons were presented at *δ* = 160.1, 144.1, 79.0. COSY experiment of **1** exhibited three spin coupling systems, CH_3_-CH=C (Frag. A), CH=CHCH-O (Frag. B), and CH_2_CH_2_CHOH (Frag. C). HMBC correlations observed between protons at *δ* = 1.75 (CH_3_ in Frag. A), *δ* = 6.80 and 6.21 (CH=CH in Frag. B) and a quaternary olefinic carbon at *δ =* 144.1 showed Frag. A and Frag. B were linked through the quaternary olefinic carbon. Thus, fragment D was deduced (Frag. D). The key HMBC correlations between three olefinic protons at *δ* = 5.44, 6.21 and 6.80 in fragment D, an oxygenated methine proton at *δ =* 5.50 in fragment D, one methylene proton at *δ =* 2.05 in fragment C, and another oxygenated methine proton at *δ =* 4.11 in fragment C and quaternary carbon at *δ* = 79.0 suggested both ends of fragment D and one end of fragment C were connected with the same quaternary carbon *δ* = 79.0. Therefore, a fragment E was built up as shown in Figure 1. The same molecular formula and similar NMR data suggested **1** was related to streptazolin, which possesses a unique tricyclic ring system embodying an internal urethane unit and an exocyclic ethylidene side chain [7] and had been found from several *Stretomyces* sp. [2,4,8]. According to the molecular formula C_11_H_13_NO_3_ of **1** and the similar NMR data with those of streptazolin, the quaternary carbon at *δ* = 160.1 was considered belonging to a carbamoyl ester moiety just like that in streptazolin. Significant HMBC correlations between oxygenated methine proton at *δ* = 5.50, and methylene protons at *δ* = 3.58 and 3.11 and the acyl carbon at *δ* = 160.1 indicated the oxygen atom of carbamoyl ester unit was connected with the terminal carbon at *δ* = 82.6 in fragment E, and the nitrogen atom of the carbamoyl ester unit was connected with the terminal carbon at *δ* = 42.9 in fragment E. Thus, another terminal carbon at *δ* = 79.0 in fragment E was inevitably linked with the nitrogen atom of the carbamoyl ester moiety, which was confirmed by HMBC correlations between one methylene proton at *δ* = 3.58 and the quaternary carbon at *δ* = 79.0. Therefore, the planar structure of **1** was elucidated as shown in Figure 1 and named isostreptazolin. NOE correlations between H-6 (*δ* = 6.80) and H-13 (*δ* = 1.75); H-2 (*δ* = 5.44) and H-10a (*δ* = 2.48) suggested the double bond at Δ^7,12^ has a *Z* conformation. NOE correlations between H-4 (*δ* = 5.50) and H-9 (*δ* = 4.11) indicated the *cis* configuration of H-4/H-9. Just like streptazolin, **1** possesses an unusual ring system, leading the authors to postulate that **1** is related to streptazolin, and the two compounds originate from a similar biosynthetic pathway or derive from the same biosynthetic polyketide precursor [6]. 

**Figure 2:**
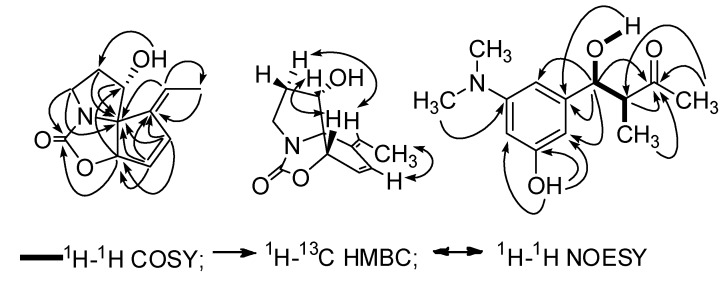
Significant HMBC and NOE correlations of **1** and COSY and HMBC correlations of **2**.

The molecular formula of **2** was assigned as C_13_H_19_NO_3_ by HRESIMS at *m/z* 238.1442 [M+H]^+^ (calcd for C_13_H_20_NO_3_, 238.1443). The IR spectrum indicated the presence of hydroxyl (3395 cm^−1^) and carbonyl groups (1703 cm^−1^) and a benzene ring (1613, 1510, 1445cm^−1^). The ^1^H NMR spectrum of **2** (Table 1) showed three aromatic proton signals at *δ* = 6.14 (1H, brs), 6.09 (1H, brs), and 5.98 (1H, brt, *J* =1.8), indicating a *meta* trisubstituted benzene ring moiety exists in **2**. Two substituents could be clearly determined as a phenolic hydroxyl by a proton signal at *δ* = 8.97 (1H, brs), and a dimethylamino group according to proton signal at *δ* = 2.83 (6H, s) and the corresponding carbon signal at *δ* = 40.0 in the ^1^H- and ^13^C-NMR of **2**. Complete assignments of the ^1^H- and ^13^C- NMR spectra of **2** were achieved with 1D and 2D NMR experiments (Table 1). Partial structures were assembled by interpretation of COSY data and connected by analysis of the HMBC NMR data. The key correlations are shown in Figure 2. As not enough sample was available, the authors could not determine the stereochemistry of C-7 and C-8 by chemical methods. Therefore, the planar structure of **2** was elucidated as 4-(3-(dimethylamino)-5-hydroxyphenyl)-4-hydroxy-3-methylbutan-2-one, and it was named sannaphenol.

**Table 1:** ^1^H- (300 MHz) and ^13^C-NMR (75 MHz) data of compounds **1** and **2** (in DMSO-*d*_6_).

No.	1		2	
	δ_C_	δ_H_	δ_C_	δ_H_
1	-	-	145.7	-
2	160.1	-	101.7	6.14 1H, brs
3	-	-	151.4	-
4	82.6	5.50 1H, d, *J* = 1.8	98.2	5.98 1H, brt, *J* = 1.8
5	131.8	6.21 1H, brd, *J* = 5.8	157.7	-
6	134.1	6.80 1H, d, *J* = 5.8	101.8	6.09 1H, brs
7	144.1	-	73.2	4.75 1H, t, *J* = 4.8
8	79.0	-	53.9	2.72 1H, m
9	72.8	4.11 1H, brt, *J* = 4.0	210.2	-
10	35.6	2.48 1H, m	28.9	2.05 3H, s
		2.05 1H, m		
11	42.9	3.58 1H, ddd, *J* = 11.0, 9.2, 7.3	10.5	0.88 3H, d, *J* = 6.6
		3.11 1H, ddd, *J* = 11.0, 9.9, 4.0		
12	118.9	5.44 1H, q, *J* = 7.2	40.0	2.83 3H, s
13	14.6	1.75 3H, d, *J* = 7.2	40.0	2.83 3H, s
3-OH				8.97 1H, brs
7-OH				5.19 1H, d, *J* = 4.8
9-OH		5.47 1H, d, *J* = 4.0		

### 2.2. Biological Activity

Streptazolin showed no inhibition against several human cancer cell lines, while its precursors [9] and derivatives [4,10] indicated significant cytotoxic activity. In this paper, the cytotoxic effects of **1** and **2** were tested against human large-cell lung carcinoma cell line (H460) and human cervix carcinoma cell line (HeLa). Both compounds were inactive against these two cell lines at 100 μM (inhibition ratio lower than 50%).

## 3. Experimental

### 3.1. General

Optical rotations were determined using a SGW-1 automatic polarimeter (Shanghai Precision & Scientific Instrument Co., Ltd). IR spectra were recorded with Nicolet Avatar FT-IR spectrometer (KBr). NMR spectra were recorded on Bruker ARX-300 and Bruker AV-600 spectrometers. ESIMS were recorded by Finnigan LCQ mass spectrometer. HRESIMS were measured with QSTAR LCQ mass spectrometer. Ultraviolet spectra were performed with a Waters 2695 separation module and Waters 2996 photodiode array detector. Solid-phase extraction was done on polymeric resin Amberlite XAD-16 (Rohm & Hass, France). Silica gel (100–200 mesh, 200–300 mesh, Qingdao Marine Chemical Ltd., Qingdao, China), Sephadex LH-20 (Amersham Biosciences), and YMC*GEL ODS-A (S-50 μm, 12 nm) (YMC Co., Ltd, Japan) were used for column chromatography. MTT assay was recorded on a microplate reader (KHB ST-360, SH Kehua Laboratory System Co., Ltd., Shanghai, China).

### 3.2. Producing Organism

The organism *Streptomyces sannanensis* and fermentation conditions were identical to those in reference [1].

### 3.3. Extraction and Isolation

The completed fermentation broth (80 L) was separated into filtrate and mycelium by centrifugation. The culture filtrate was absorbed onto the polymeric resin Amberlite XAD-16. The salt and high molecular materials were washed out with water, and then, other absorbed organic material was eluted with MeOH to yield 55 g of dried extract after removing the solvent under vacuum. The dried extract was extracted with petroleum, CHCl_3_ and EtOAC respectively, the CHCl_3_ extract fraction (2 g) was subjected to gel chromatography on Sephadex LH-20 (MeOH) to produce three fractions. Fraction 2 (1.4 g) was resubmitted to gel chromatography on Sephadex LH-20 (MeOH) to produce seven fractions (Fr.2.1-Fr.2.7). Fr.2.4 (550 mg) was then separated by silica gel column chromatography (hexane-EtOAc = 5:2-1:1-EtOAc) and yielded eight subfractions (Fr. 2.4.1–2.4.8). Fraction 2.4.6 was purified by silica gel column chromatography (hexane-EtOAc = 2:1) to give compound **2** (13 mg). Fraction 2.4.7 was separated by ODS column chromatography, eluting with water-methanol (4:6) yielding compound **1** (9.8 mg).

### 3.4. Compound Characterization

*Isostreptazolin* (**1**): colorless oil. [α]^20^_D_ + 48.9 (*c* 0.45, MeOH). UV (MeOH) λ_max_: 236 nm. IR (KBr) ν_max _3366, 2958, 2922, 1718, 1635, 1382, 1236, 1045 cm^−1^. ESI-MS *m/z*: 208 [M+H]^+^, 230 [M+Na]^+^, 437 [2M+Na]+, 206 [M-H]−, 242 [M+Cl]−, 413 [2M-H]−, 449 [2M+Cl]−. HRESI-MS *m/z*: 208.0966 [M+H]^+^ (calcd for C_11_H_14_NO_3_, 208.0974). ^1^H- and ^13^C-NMR data see Table 1.

*Sannaphenol* (**2**): colorless oil. [α]^20^_D_ + 34.3 (*c* 0.60, MeOH). UV (MeOH) λ_max_: 215, 253, 295 nm. IR (KBr) ν_max _3395, 2930, 1703, 1613, 1592, 1510, 1485, 1445, 1366, 1244, 1152, 1017 cm^−1^. ESI-MS *m/z*: 238 [M+H]^+^, 260 [M+Na]+, 497 [2M+Na]+, 236 [M-H]−, 272 [M+Cl]−, 473 [2M-H]−, 509 [2M+Cl]−. HRESI-MS *m/z*: 238.1442 [M+H]^+^ (calcd for C_13_H_20_NO_3_, 238.1443). ^1^H- and ^13^C-NMR data see Table 1.

### 3.5. Cytotoxic Assay

The human large-cell lung carcinoma cell line (H460) and human cervix carcinoma cell line (HeLa) were used to evaluate cytotoxic effects of **1** and **2** with an MTT method [11]. H460 was grown in RPM1-1640 medium plus 10% heat-inactived fetal bovine serum and HeLa was grown in DMEM medium plus 10% heat-inactived fetal bovine serum. The assays were performed in 96-well microtiter plates. Compounds **1** and **2** was dissolved in DMSO and diluted to six different concentations (10, 3.3, 1.0, 0.33, 0.10, and 0.033 mM), and each solution was ten-fold diluted to six different concentations using culture medium (1.0, 0.33, 0.10, 0.033, 0.010, and 0.0033 mM), then each solution (10 µL) was added to culture medium wells (90 µL, about 5000 cells). After incubation at 37 °C for 72 hours, MTT (10 µL, 5 mg/mL) was added to each well and incubated for four hours, and then liquid in the wells was removed. DMSO (150 µL) was added to each well. The absorbance was recorded on a microplate reader at a wavelength of 570 nm.

## 4. Conclusions

Two new metabolites have been isolated from *Streptomyces sannanensis*. The new compounds were spectroscopically identified and given the trivial names isostreptazolin and sannaphenol. Both the new compounds were inactive against the H460 and HeLa cell lines at 100 μM.
